# Ultrasound-Guided Genicular Nerve Thermal Radiofrequency Ablation for Chronic Knee Pain

**DOI:** 10.1155/2016/8292450

**Published:** 2016-10-16

**Authors:** Joshua Wong, Nicholas Bremer, Paul D. Weyker, Christopher A. J. Webb

**Affiliations:** ^1^Columbia University Medical Center, Department of Anesthesiology, New York, NY, USA; ^2^Divisions of Pain Medicine, Regional Anesthesia & Critical Care Anesthesia, Columbia University Medical Center, Department of Anesthesiology, New York, NY, USA; ^3^Divisions of Pain Medicine, Regional Anesthesia & Liver Transplant Anesthesia, Columbia University Medical Center, Department of Anesthesiology, New York, NY, USA; ^4^Kaiser Permanente, San Francisco, CA, USA; ^5^Anesthesiology, Perioperative & Pain Medicine, Stanford University School of Medicine, Stanford, CA, USA

## Abstract

Osteoarthritis (OA) of the knee is one of the most common joint diseases affecting adults in the United States. For elderly patients with multiple medical comorbidities who do not wish to undergo total knee arthroplasty (TKA), lifestyle modification, pharmacologic management, and injections are the mainstay of therapy. Previously, pain management interventions were limited to intra-articular joint injections and viscosupplementation with hyaluronic acid. Fluoroscopic-guided techniques for radiofrequency ablation (RFA) of the genicular nerves have been previously described and a recent cadaveric study suggests that ultrasound-guided genicular nerve blocks can be performed accurately. We performed an ultrasound-guided radiofrequency ablation of the genicular nerves in 88-year-old woman who had deferred surgical management given her age. Following successful ultrasound guided diagnostic genicular nerve blocks, she proceeded to RFA using the same ultrasound guided technique. The procedure resulted in significant pain relief and improvement in overall function for greater than 6 months. The use of ultrasound provides a relatively rapid and noninvasive method to directly visualize genicular nerves and surrounding vasculature. Our case suggests that, for genicular nerve blockade and RFA, ultrasound may be a useful alternative to fluoroscopy. Not only did the procedure result in significant pain relief that has persisted for greater than 6 months but also more importantly her function status and quality of life were improved.

## 1. Introduction

Osteoarthritis (OA) of the knee is one of the most common joint diseases affecting adults in the United States [1]. Symptomatic OA clinically manifests as either pain or decreased function and affects roughly 10% of men and 13% of women over the age of 60. Population studies have shown that the prevalence of symptomatic OA is around 20% in individuals greater than 65 years of age [2]. Diagnosis of OA is made when the clinical signs and symptoms correlate with radiologic changes. For elderly patients with multiple medical comorbidities who do not wish to undergo total knee arthroplasty (TKA), lifestyle modification, pharmacologic management, and injections are the mainstay of therapy. Previously, pain management interventions were limited to intra-articular joint injections and viscosupplementation with hyaluronic acid [3]. Recently, Choi et al. described a fluoroscopic-guided technique for radiofrequency ablation (RFA) of the genicular nerves [4]. Our case describes an ultrasound-guided technique for genicular nerve blockade and RFA, the first of its kind reported in the literature.

## 2. Case Description

An 88-year-old woman with osteoporosis and chronic joint pain presented to the CUMC pain clinic for assistance in managing her chronic knee pain. Her 10-point visual analogue scale (VAS) for pain was 8/10 with activity and 3/10 at rest. She was previously treated with a series of three intra-articular knee injections, which helped her pain for approximately one month. She was also prescribed acetaminophen and diclofenac 1% gel but could not take oral nonsteroidal anti-inflammatory drugs due to a history of severe gastritis. A recent knee X-ray demonstrated severe medial femorotibial and mild lateral femorotibial compartment osteoarthrosis of the right knee. Physical exam during this visit was significant for bilateral mild knee edema, crepitus, and pain with flexion/extension of both knees. The patient was not interested in surgery and was referred from her orthopedic surgeon for pain management.

**Figure 1:**
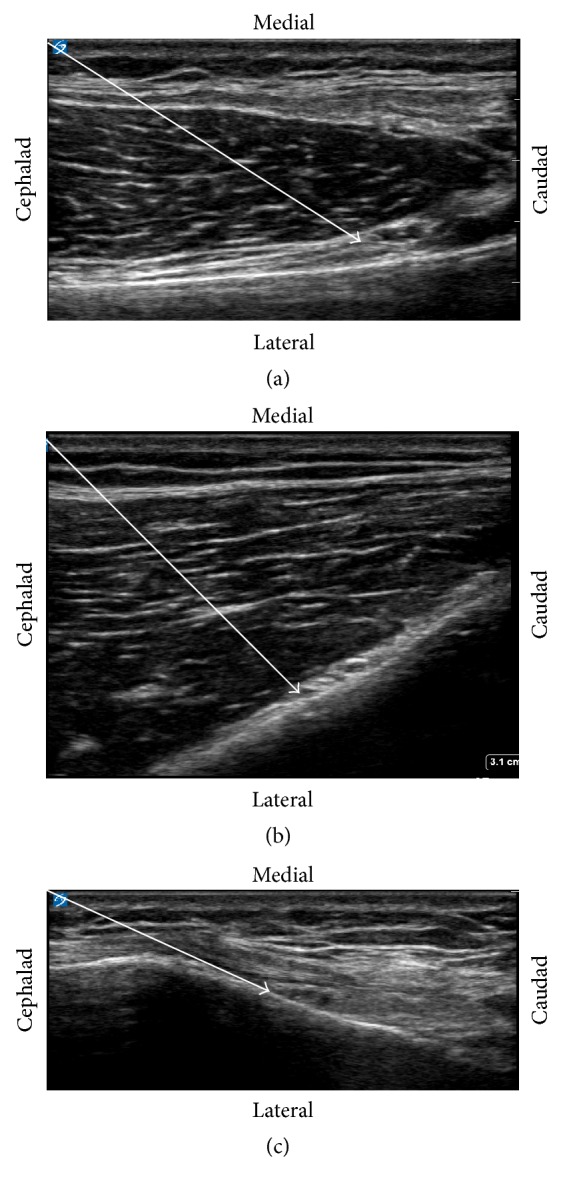
For all procedures, 22 G, 5 mm active tip, 50 mm long RF needle was advanced to the target area under continuous ultrasound guidance. Impedance was between 400 and 500 ohms. Sensory testing was positive up to 0.40 mV, 50 Hz, described as a pressure-like sensation which was concordant with the patient's usual distribution of pain. Motor testing up to 3 mV at 2 Hz was negative. After stimulation and negative aspiration, 2.0 cc of 2% Lidocaine was injected. At this point, continuous radiofrequency lesioning was started at 80°C for 90 sec. The needle was then removed and the area dressed. (a) Superomedial genicular nerve (SMGN): a high frequency, linear ultrasound (Sonosite, Bethel, WA, USA) transducer was placed in the sagittal orientation over the right femoral medial epicondyle and translated proximally to the level of the adductor tubercle and the insertion of the adductor magnus tendon. The bony cortex one cm anterior to the peak of the adductor tubercle was targeted for the injection. The presence of the SMGN's corresponding artery confirmed the target area. Using an in-plane technique, the needle was inserted in a cephalad to caudad direction. The solid white arrow delineates the needle trajectory. (b) Superior lateral genicular nerve (SLGN): a high frequency, linear ultrasound (Sonosite, Bethel, WA, USA) transducer was placed in the sagittal orientation over the right femoral lateral epicondyle and translated proximally to the level of the insertion of the biceps femoris tendon. The bony cortex was targeted near the SLGN's corresponding artery. Using an in-plane technique, the needle was inserted in a cephalad to caudad direction. Solid white arrow delineates the needle path. (c) Inferior medial genicular nerve (IMGN): a high frequency, linear ultrasound (Sonosite, Bethel, WA, USA) transducer was placed in the sagittal orientation over the right tibial medial epicondyle. The medial collateral ligament was visualized. The transducer was then translated distally to the level of the tibial insertion site of the medial collateral ligament below the tibial medial epicondyle. The point of the bony cortex at the midpoint between the peak of the tibial medial epicondyle and the initial fibers inserting on the tibia of the medial collateral ligament was targeted for the injection to the IMGN. The presence of the IMGN's corresponding artery confirmed the target area. Using an in-plane technique, the needle was inserted in a cephalad to caudad direction. The solid white arrow delineates the needle path.

Our plan was for a diagnostic genicular nerve block, which—if successful—would be followed by a continuous RFA lesion. The patient underwent ultrasound-guided nerve block with local anesthetic of the right superomedial genicular nerve (Figure 1(a)), superolateral genicular nerve (Figure 1(b)), and inferomedial genicular nerve (Figure 1(c)). Two milliliters of 0.5% ropivacaine was injected at each location. The patient reported significant improvement in pain (VAS scores of 2/10 with activity and 0/10 at rest) and function with this block and was scheduled for continuous RFA the following week. At the one-month follow-up visit, the patient had complete pain relief with VAS pain scores of 0/10 with activity and 0/10 at rest. Functionally, she was able to walk around home and to the store without limitations. At six months, she continues to be 100% pain-free (VAS pain scores ranging from 0 to 2 with activity and 0/10 at rest) without any functional limitations.

## 3. Discussion

Osteoarthritis of the knee is a common health problem that affects more than 20% of adults older than 65. Genicular nerve RFA has been investigated as a nonsurgical alternative in the management of chronic knee osteoarthritis [4, 5]. In the study by Choi et al., significant pain relief was seen in 60% of patients at a 6-month follow-up. In previous studies, the genicular nerves innervating the knee are targeted using a landmark approach with fluoroscopic guidance. A recent cadaveric study suggests that ultrasound-guided genicular nerve blocks can be performed accurately [6]. In our case study, we performed an ultrasound-guided radiofrequency ablation of the genicular nerves that resulted in significant pain relief that has persisted for 6 months. Using the approach described by Yasar and colleagues [6] combined with the initial landmark description by Choi et al. [4] we identified the genicular nerve with ultrasound by first localizing their arterial branches. The nerves were then followed cephalad and caudad as they pierced through the overlying muscle layers. The use of ultrasound provides a relatively rapid and noninvasive method to directly visualize genicular nerves and surrounding vasculature. Our case suggests that, for genicular nerve blockade and RFA, ultrasound may be a useful alternative to fluoroscopy.
